# Immunometabolic Reprogramming in Hepatocellular Carcinoma Mechanisms, Biomarkers, and Therapeutic Implications

**DOI:** 10.32604/or.2026.080746

**Published:** 2026-06-16

**Authors:** Guodong Yu, Weiying Ge, Hongliang Yao, Xuefeng Bai, Jianfei Wu, Yuan Wang, Jiangtao Bai, Yong Cui, Jijing Han

**Affiliations:** 1 Department of Hepatobiliary Surgery, Affiliated Hospital of Hebei University, Baoding, China; 2 Department of Radiology, Affiliated Hospital of Hebei University, Baoding, China; 3 The Second Department of general surgery, Hengshui people’s Hospital, Hengshui, China; 4 Department of Interventional and Vascular Surgery, Affiliated Hospital of Hebei University, Baoding, China; 5 Department of Pediatrics, Affiliated Hospital of Hebei University, Baoding, China

**Keywords:** Metabolic reprogramming, tumor microenvironment, biomarkers, immune checkpoint, hepatocellular carcinoma

## Abstract

Hepatocellular carcinoma (HCC) develops in a chronically inflamed and dysregulated liver metabolism, in which tumor progression and resistance to treatment are orchestrated by the changes in cellular metabolism and immune control. Growing evidence recognizes immunometabolic reprogramming as the two-way interaction of metabolic processes and immune cell capabilities as one of the major determinants of immune evasion and heterogeneity of treatment response in HCC. The review aims to comprehensively evaluate immunometabolic reprogramming in hepatocellular carcinoma, with a focus on its role in tumor progression, immune regulation, and its potential for biomarker identification and therapeutic targeting. Dysregulated glycolysis, lipid metabolism, amino acid utilization, and mitochondrial dysfunction contribute to remodeling of the tumor microenvironment and defects in antitumor immunity. Immunometabolic biomarkers derived from tumor tissue, immune cell states, circulating and liquid biopsy platforms, and metabolic imaging are critically examined for their clinical relevance and associations with disease outcomes and treatment responses. Besides, the role of different immunometabolic conditions on therapeutic efficacy, specifically within the frames of immune checkpoint-inhibitor-based and combination regimens, is addressed. Altogether, immunometabolic reprogramming is identified as a common framework of biomarker-based stratification and precision therapeutic techniques in hepatocellular carcinoma.

## 1 Introduction

Metabolic regulation is primarily governed by the largest gland in the human body, the liver, which plays an important role in maintaining metabolic homeostasis and influences disease progression [1]. Liver maintains the metabolism of both micronutrients and macronutrients, including xenobiotics, bile, hormones, carbohydrates, lipids, and proteins [2]. However, several risk factors like hepatitis B and C, NAFLD (non-alcoholic fatty liver disease) disturb the normal hepatic architecture [3]. This may lead to cirrhosis, fibrosis, and chronic liver inflammation that may cause abnormal metabolic remodeling and liver tumor development [4]. In addition, the mutations in oncogenes and tumor suppression genes, including p21, p53, and RAS, the development of hepatocellular carcinoma (HCC), is facilitated by repeated regenerative repair of hepatocytes [5]. Hepatocellular carcinoma represents the most common type of primary liver cancer and represents a major global health burden [5,6]. The study Bridge to Better Outcomes in HCC (BRIDGE) proposes that 64% percent of advanced HCC in China is no longer a radical treatment case [7]. Tumor stage is another important prognostic variable of HCC, and fewer than 20% percent of the patients could be detected at the early stage because of the predictive ability and other considerations [8]. In the case of local HCC, the five-year overall survival rate was approximately 32.6%, and in the case of metastatic HCC, it was not more than 2.4% [9]. Early HCC is commonly treated with tumor excision, local percutaneous ablation including radiofrequency ablation, and liver transplantation [10]; however, the recurrence rate of this disease is very high, with a 5-year recurrence rate lying in the range of 40–70% years, and no method of reducing the likelihood of the disease reoccurring is known [11]. Their frequent hypoxic and hypo-nutrient states mean that cancer cells must adapt their requirements to high-energy and other biomaterials when making biomass [12]. Some of the processes that are influenced include increased glycolysis, synthesis of fatty acids and nucleotides, and metabolism of amino acids, among others. This is termed as metabolic reprogramming [13] and is characteristic of cancer that renders it resistant to medications, metastatic, and progressive. Apart from sustaining malignant cell proliferation, metabolic reprogramming performs an important role within the tumor microenvironment (TME) [14].

The Warburg effect is the tendency of tumor cells to prefer the glycolysis process at the expense of the oxidative phosphorylation (OXPHOS) process, even in the presence of oxygen, as opposed to normal cells [15]. The rate of production of the ATP of aerobic glycolysis is much greater than that of the OXPHOS, which is more effective in supporting the avaricious reproduction of neoplastic cells, even though aerobic glycolysis is much less efficient than OXPHOS (2 ATP vs. 36 ATP per glucose) [16]. Nevertheless, surplus lactate from aerobic glycolysis drives neighboring oxygenated cells, inducing a symbiosis in metabolism between oxidative and glycolytic metabolism [17]. Glucose (GLC) bulk tissues have a significant absorption of glucose, and the PET (Positron Emission Tomography) scan with the use of 18F-2-deoxyglucose (18FDG) is commonly used to diagnose and monitor the progression of liver cancer [18]. Also, other central metabolic programs are often reprogrammed, leading to dysregulated nutrient depletion, accumulation of oncometabolites, and signaling pathway imbalances in the TME, in addition to glucose metabolism [19]. It is increasingly becoming known that the blood supply to the intestine transports gut bacterial metabolites to the liver. The alteration of the gut microbiota modulates immune cell infiltration and activity and has an influence on the efficacy of immunotherapy in the TME of liver cancer [20]. Tumors may thus be considered to be metabolic disorders, and it may be possible to improve the treatment outcome of tumors by modulating the glucose metabolism of the immune cells, as well as the tumor cells [21]. Recent changes in systemic therapy, including immune checkpoint inhibitors and combo regimens, have not been able to establish uniform clinical outcomes in hepatocellular carcinoma [22]. Conventional predictive markers based on tumor burden, staging system, and immune checkpoint expressions have shown limited reliability, and only a subset of patients derive durable clinical benefit [23]. According to emerging clinical and translational evidence, the metabolic constraints of the tumor microenvironment should be considered to achieve a correct understanding of immunological dysfunction in hepatocellular carcinoma [23]. The combination of immune cell metabolic depletion and tumor-intrinsic metabolic reprogramming determines anticancer immunity and influences the effectiveness of the therapy [24]. Thus, immunometabolic reprogramming in hepatocellular carcinoma is an important phenomenon in the disease and in the response to treatment, rather than a secondary phenomenon [25]. Metabolic alterations within tumor cells and immune populations play an important role in tumor progression and therapeutic effects in HCC [26]. Changes in glucose, lipid, and amino acid metabolism result in tumor growth and therapeutic resistance within TME [27]. Understanding the molecular mechanisms underlying these metabolic adaptations is necessary for improving clinical management, identifying reproducible biomarkers, and developing effective therapeutic techniques [28].

This review provides an evidence-based review of immunometabolic reprogramming in hepatocellular cancer that is comprehensive and has a focus on proven processes, clinically significant biomarkers, and treatment outcomes. Therefore, this review aims to systematically evaluate immunometabolic reprogramming in HCC, with special emphasis on its role in tumor growth, immune modulation, and its potential for biomarker identification and therapeutic targeting. In hepatocellular carcinoma, immunometabolic reprogramming demonstrates the dynamic crosstalk between tumor intrinsic metabolic changes and the immune microenvironment. Variations in glycolysis, lipid metabolism, amino acid consumption, and mitochondrial function not only promote tumor growth but also have an impact on immune cell infiltration, stimulation, and exhaustion within the tumor microenvironment [29]. This comprehensive perspective is necessary for understanding disease progression, clinical response, and the development of biomarkers in hepatocellular carcinoma.

**Figure 1:**
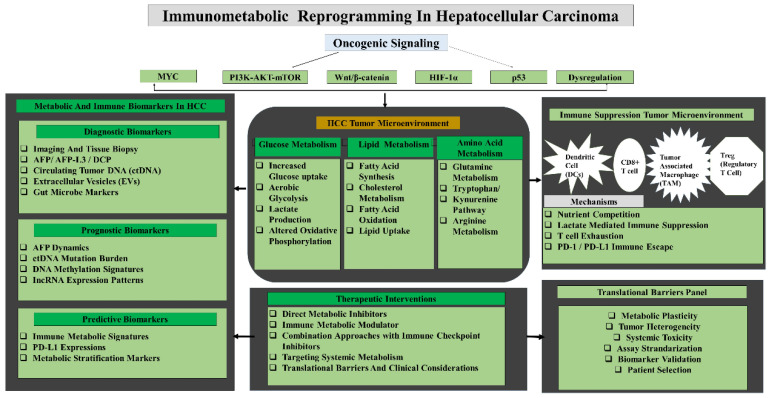
Conceptual framework of immunometabolic reprogramming I hepatocellular carcinoma (HCC). Where MYC is MYC proto-oncogene, PI3K/AKT-mTOR is phosphoinositide 3-kinase-AKT-mechanistic target of rapamycin, Wnt/β-catenin is Wingless-related integration site/β-catenin signaling pathway, HIF-1α is hypoxia-inducible factor 1-alpha, p53 is tumor protein p53, DCs is dendritic cells, CD8^+^ T cells is cluster of differentiation 8 positive T cells, TAM is tumor associated macrophages, Treg is regulatory T cells, PD-1 is programmed cell death protein, PD-L1 is programmed death-ligand 1.

The conceptual relationships among the oncogenic pathways, metabolic reprogramming, immune suppression, biomarker development, and therapeutic intervention strategies in hepatocellular carcinoma are summarized in Fig. 1.

## 2 Immunometabolic Landscape of Hepatocellular Carcinoma

HCC is known to occur in chronically inflammatory liver, in which immune cells are functionally reprogrammed with inappropriate immune responses resulting in an environment that is permissive to malignancy [30]. These immunological changes make it possible to develop deformed cells, and this leads to the development of HCC [31]. Therefore, it is reasonable to believe that the quantity of the invading immune cells, their composition, and the level of development influence the prognosis of HCC and their ability to respond to pharmaceutical interventions [32]. MDSCs (myeloid-derived suppressor cells) play a dominant role in immune suppression and consist of a diverse array of immature myeloid cells within the TME. The MDSCs have been shown to mediate T-cell proliferation suppression in patients with HCC by depleting arginine due to high levels of arginase activity [33]. Moreover, the production of matrix metallopeptidase 9 (MMP-9) by MDSCs enhances neoangiogenesis by raising the bioavailability of vascular endothelial growth factor (VEGF). The MDSCs can release Interleukin-10 (IL-10) that leads to M2 polarization, which suppresses CD4^+^ and CD8^+^ lymphocytes, natural killer (NK) cells, and β (TGF-β). Tumor-associated macrophages (TAMs) are another common cellular component of the HCC immunological milieu [34]. TAMs are a subpopulation of macrophages that regulate the malignant development of HCC and produce diverse molecular mediators such as programmed death-ligand 1 (PD-L1), transforming growth factor beta (TGF-β), and IL-10 [33,35]. Specifically, TAMs stimulate angiogenesis, cancer metastasis, and epithelial-mesenchymal transition (EMT) [36]. They also repress T-cell activation and attract neutrophils, which are critical in the evolution of hepatic carcinogenesis. Recent findings have shown that there are tumor-associated neutrophils (TANs) that are immunosuppressive in nature [36,37]. TANs sustain the development of HCC by expressing PD-L1, which links the programmed cell death protein PD-1 as an immunological checkpoint on T cells and sends a signal of inhibition. TANs secrete oncostatin M (OSM) and promote the metastasis of tumors [38]. Moreover, the growth and expansion of Tregs (immunosuppressive Foxp3^+^ regulatory T cells) found in tumoral tissue, suppression of anti-cancer immunity, and effector T cell functions are also regulated by TANs [39]. TILs are tumor-infiltrating leukocytes that play a role in anticancer responses by having a direct impact on the innate response or angiogenesis, and the adaptive immune system [40]. Tregs (CD4^+^) are another type of cell that can potentially boost immunological tolerance and block the anticancer activity of other cells [41]. Excessive exposure of antigens to the lymphocytes (CD8^+^) results in the production of CD8^+^ cells that are referred to as exhausted CD8^+^ cells, which have lost their ability to destroy the cells [41]. Exhausted CD8^+^ cells exhibit functional decline characterized by reduced cytokine secretion alongside constant upregulation of immune inhibitory receptors such as PD-1, CTLA-4, and related checkpoint molecules [42]. The simultaneous inhibition of antitumor immunity by plasma cell proliferation within the microenvironment of HCC is caused by the production of PD-L1 and IL-10, inhibitory mediators, and by the inhibition of CD8^+^ T-cell activation [31]. These immunosuppressive associations within the tumor microenvironment are closely linked to the metabolic changes in HCC, emphasizing the importance of immune metabolic reprogramming in disease progression and therapeutic effects [29]. Moreover, these immune populations also depict distinct metabolic phenotypes that promote tumor growth. Tumor-linked macrophages often rely on increased fatty acid oxidation and oxidative metabolism, facilitating their pro-tumorigenic activity [43]. Myeloid-derived suppressor cells show increased glycolysis and lipid metabolism, which facilitates their expansion and suppressive capacity [44]. Regulatory T cells preferentially use fatty acid oxidation and mitochondrial metabolism to sustain their stability within the tumor microenvironment. In contrast, exhausted CD8^+^ T cells experience metabolic insufficiency characterized by impaired glycolysis and mitochondrial dysfunction, leading to reduced cytotoxic activity against tumor cells [45].

## 3 Mechanism

Several molecular processes that regulate cell death and proliferation are associated with liver carcinogenesis. In experimental studies, structural genomic changes were observed in the initial phases of liver carcinogenesis [46]. HBV hepatocyte genome integration leads to genomic instability, Ras and β-catenin signaling transactivation, and Ras and β-catenin rearrangement [47]. There is an upregulation of insulin-like growth factor-2 (IGF-2) and TGF-α by the HCV core protein [48]. The genetic variations in HCC involve three major pathways: (i) the p53-dependent, (ii) the RB1 (retinoblastoma-1)-dependent, and (iii) the Wnt-dependent [49]. The Wnt proteins bind to specific Frizzled receptors on the cell surface of target cell, activating various intracellular pathways [50]. This results in the accumulation and nuclear localization of the β-catenin protein, which is a sign of activation of the canonical Wnt pathway and acts on specific genes like survivin, c-Myc, and cyclin D1, which are critical in the formation of cancer [50,51]. Clinical studies suggest that the aberrant activation of the Wnt/β-catenin cascade is highly linked with liver carcinogenesis [52]. Approximately 33–68 percent of HCC tissues accumulate β-catenin in their nucleus and cytoplasm, whereas the corresponding normal tissues lack any accumulation of the protein [53]. SiRNA knockdown of the Frizzled-7 receptor abolished Wnt/β-catenin signaling activation [54]. More importantly, co-immunoprecipitation experiments demonstrated the Wnt3-Frizzled-7 receptor interaction, which showed that the Frizzled-7 receptor regulated the action of Wnt3 [54]. Proteomics findings suggest that elevated Wnt-1 levels linked with nuclear Factor kappa-light-chain-enhancer of activated B cells (NF-kβ) can be a central process in liver carcinogenesis in HCC [55].

Signals transmitted to the MAPK cascade are sent by EGFR, a tyrosine kinase receptor; PDGFR (platelet-derived growth factor); VEGFR (vascular endothelial growth factor), and HGF/MET, known as hepatocyte growth factor [56]. There is an activation of MEK-1/2, ERK-1/2, and RAF-1 by Active Ras (Ras-GTP) [57]. ERK1/2 activation or phosphorylation activates many growth-related genes, including cJUN, c-FOS, c-MYC (proliferation and survival mechanisms), HIF-1α (hypoxia-induced factor), VEGF (vascular endothelial growth factor), and HKII (Hexokinase II) [58]. Persistent activation of ERK1/2 causes large cell proliferation in the absence of growth factors. This disease may cause tumor progression [59]. Genes associated with the MAPK cascade, such as c-FOS, c-JUN, c-RAF, and Ras-GTP, can be overexpressed in rodent-induced HCC [60]. Rat and human liver lesions show an increase in the expression of the 3-hydroxy-3methylglutaryl-CoA reductase gene, which encodes a crucial enzyme in the *de novo* production of the precursor of isoprenoid residues such as mevalonate, to activate Ras [61]. Recent studies indicate that HCC contains high concentrations of active Ras and the lowest concentration of active pMEK-1/2 and RAF-1 [61]. This coincides with a large upregulation of RAF-1 and MEK-1/2 phosphorylation/activation inhibitors, i.e., RAF kinase inhibitory protein (RKIP) and disabled homolog 2 (Dab2) [62]. The main mediators of the cascade (B-RAF and H-ras) are up-regulated in HCC, which implies their role in malignancy [63]. The mechanisms that explain Ras signaling in HCC can be categorized as follows: (i) Overexpression of H-ras; (ii) amplification of the number of DNA copies in the B-RAF genomic locus (chromosome 7q34); and (iii) epigenetic signaling, including the methylation of RASSF1A and NORE1A tumor suppressor genes [63].

The Ras-Raf-ERK-dependent pathway is associated with the molecular pathophysiology of HCC through three mechanisms: (i) the Ras protein is stimulated in 30% of HCC cases; (ii) the Raf kinase is overexpressed in the majority of HCC cases; (iii) a variety of upstream growth factors such as EGF, VEGF, PDGF, and TGFa can activate the pathway by binding to tyrosin kinase receptors [64]. Recent advances in technologies such as DNA microarrays and other molecular profiling techniques have enabled new insights into the molecular genetics of HCC to be made possible [65]. Molecular profiling has successfully identified potential HCC-associated genes either related to metastatization (NM23-H1, osteopontin, RhoC, KAI1, MMP14), tumor growth (p16, SOCS1, PEG10), or recurrence (REL, A20, vimentin, PDGFRA) [66]. Beyond their impact on tumor growth, oncogenic pathways such as Wnt/β-catenin and Ras-Raf-ERK also facilitate metabolic reprogramming in HCC. Stimulation of these pathways enhances metabolic changes, including increased glycolysis, fatty acid synthesis, and altered nutrient utilization that promote tumor growth [67]. These metabolic changes can transform the tumor immune microenvironment by restricting the availability of nutrients for effector immune cells and facilitating immunosuppressive signaling. Hence, oncogenic pathway-driven metabolic reprogramming may enhance immune exclusion and limit the effectiveness of therapeutic strategies in HCC [68].

## 4 Metabolic Reprogramming

### 4.1 Increased Glycolysis

**Figure 2:**
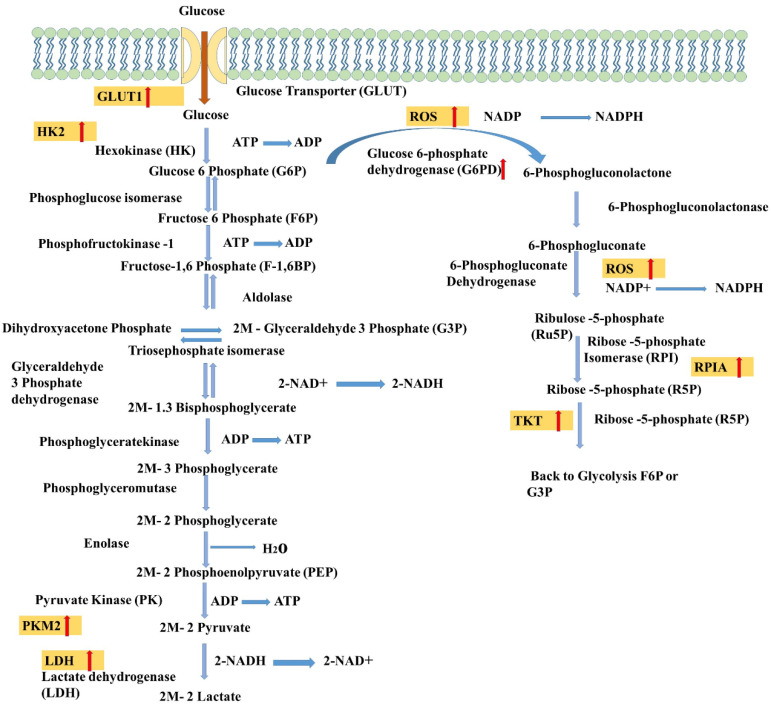
HCC rewired glycolysis and pentose phosphate pathway. The major change of glucose metabolism in HCC cells is the increase in the level of several enzymes that stimulate glucose uptake and its use. These adaptations encompass augmented levels of GLUT1 and HK2, which raise glucose uptake, PKM2, which raises augmented pyruvate formation, and LDH, which facilitates change of pyruvate to lactate that favors anaerobic glycolysis. This diagram also shows the PPP, which is an important metabolic pathway that forms R5P to create the nucleotides and NADPH to create the lipids and antioxidants. The enhancement of the PPP is usually observed in HCC, which is shown through the amplified activity of such enzymes as G6PD and TKT. Where GLUT1 is glucose transporter 1, HK2 is hexokinase 2, ROS is reactive oxygen species, G6PD is glucose-6-phosphate dehydrogenase, RPIA is ribose-5-phosphate isomerase A, TKT is transketolase, PKM2 is pyruvate kinase M2, LDH is lactate dehydrogenase, PPP is pentose phosphate pathway, R5P is ribose-5-phosphate, NADPH is nicotinamide adenine dinucleotide phosphate (reduced form), NADP+ is nicotinamide adenine dinucleotide phosphate.

In the maintenance of blood glucose levels, the liver plays an important role [2]. To achieve glucose homeostasis, the body stores excess glucose as glycogen and releases glucose into the blood to restore the normal level [69]. Glycogen catabolism is employed by the liver to release glucose when a person is fasting or when they require more energy [70]. The Warburg effect explains the extensive dependence on glycolysis to generate energy in the HCC despite the availability of oxygen [71]. This change of oxidative phosphorylation to glycolysis, which represents a typical feature of anaerobic metabolism, permits HCC cells to produce energy in the presence of oxygen. HCC exhibits enhanced uptake of glucose and metabolism due to the variable expression of the key glycolytic genes, such as hexokinase (HK), glucose transporters (GLUTs), and pyruvate kinase (PK) [72] (Fig. 2).

The GLUT1-4 family of transporters helps in the glucose uptake into the cytoplasm through the plasma membrane. The glucose that enters a cell is converted irreversibly into glucose-6-phosphate (G6P) [73]. Consequently, HCC tumors have high levels of G6P relative to the other liver tissue mediated by a group of enzymes known as HKs. HK4 is an isoform of HK found in hepatocytes [73,74]. HK2 is upregulated in patients has been linked with tumor growth and clinical stage; however, its diagnostic and therapeutic relevance in patients remains under active investigation. In aerobic glycolysis, HK2 has a more pronounced role than other HK isoforms [75]. HK2 binds to the voltage-dependent anion-selective channel protein-1 in the mitochondrial membrane and results in increased access to ATP produced by the mitochondria. Therefore, *in vivo* and *in vitro*, HK2 hepatic deletion increases cell death and inhibits carcinogenesis [76]. In the glycolysis process, the enzyme PK transforms phosphate into phosphoenolpyruvate (PEP) into ADP to yield pyruvate and ATP. PKM2, the M2 isoform of PK, has been associated tumors and tumor progression in HCC and is mostly observed in multiplying cells [77]. Lactate dehydrogenase (LDH) catalyzes the conversion of pyruvate to lactate, which is a common feature of HCC [78]. Although HK2, PKM2, and LDH are consistently associated with glycolytic reprogramming in HCC, the current strength of clinical evidence remains uneven [79]. Most available data are derived from tumor expression analysis, retrospective analysis, and preclinical models; robust perspective validation in patient cohorts is still limited. From a therapeutic approach, targeting glycolysis is biologically feasible but clinically challenging because glycolytic pathways are also necessary for normal tissues and activated immune cells, raising concerns regarding systemic toxicity and unintended disruption of antitumor immunity. Therefore, glycolysis-targeted therapies in HCC are more feasible when utilized in biomarker-guided settings or in rational combination with other therapies rather than as broadly applied monotherapy [80].

### 4.2 Activation of Pentose Phosphate Pathway

The pentose phosphate pathway (PPP) is essential to the proliferation of cancer cells. An element of oncogenic metabolic reprogramming is an increase in metabolic flux via the PPP to increase the availability of nucleotides and reduce equivalents [81]. Enhanced PPP redirects G6P to the production of NADPH and ribose 5-phosphate (R5P) as depicted in Fig. 2. Whilst NADPH is essential in the synthesis of lipids and has a role in regulating cellular reactive oxygen species (ROS) levels. G6P dehydrogenase (G6PD), a rate-limiting enzyme in PPP, oxidizes G6P to 6-phosphogluconolactone [82]. In HCC, G6PD is often overexpressed and facilitates the cellular characteristics of invasion, migration, epithelial-mesenchymal transition, and resistance to apoptosis. The regulation and activity of G6PD in HCC remain unclear [83]. Overexpression of G6PD has been demonstrated to make HCC cells more resistant to chemotherapy in clinical practice due to the activation of the ID1-WNT-β-catenin-MYC signaling cascade [84]. Moreover, mTORC1 increases the passage of materials across PPP by stimulating the glycolysis process for PPP substrates. It also elevates the levels of R5P isomerase A and G6PD [84]. The expression of G6PD is also regulated by the nuclear factor-erythroid 2-related factor 2 (NRF2). Transketolase (TKT) is another necessary PPP enzyme. NRF2 and BACH1 exert opposing regulatory effects on TKT, where NRF2 drives upregulation of TKT channels, glucose metabolites into glutathione production, contributing to resistance against oxidative stress [85]. High levels of TKT in HCC are linked with enhanced tumor severity and inadequate response to therapies with sorafenib [86]. Wnt, mTORC1, and NRF2 cascades can be targeted to improve patient outcomes in HCC, as these cascades interact intricately to regulate PPP [87]. The stimulation of the PPP pathway provides important metabolic benefit in HCC by supporting both biosynthetic demand and redox homeostasis. By generating NADPH, PPP activity aids tumor cells in maintaining antioxidant defenses and adapting to oxidative stress, while R5P production supports nucleotide synthesis and rapid proliferation [88]. In the tumor microenvironment, PPP-linked redox regulation may also have an impact on immune cell function, as inadequate redox balance can disrupt effective antitumor effects and result in immune dysfunction. However, increased PPP activity has been linked with tumor growth in HCC; its validated predictive or prognostic importance remains insufficiently established, and further clinical trials are needed before PPP-related markers can be reliably applied in daily patient stratification.

### 4.3 Tricarboxylic Acid Cycle

The TCA cycle oxidizes Acetyl-CoA to produce CO2 via a series of metabolic reactions that generate NADH and FADH2 that serve as electron donors to play a role in oxidative phosphorylation (OXPHOS) in the mitochondria [89]. It is the heart of the cell metabolism, and here the production of ATP, reductive potential, and many macromolecule precursors occurs [90]. Hepatic mitochondria are involved in many metabolic activities, including the elimination of toxic substances, gluconeogenesis, and ketogenesis [91]. Normal cells generate Acetyl-CoA by either the pyruvate dehydrogenase (PDH) complex or by β-oxidation of fatty acids when oxygen is available [92]. The Warburg effect is compatible with the finding of reduced expression of the subunit PDHA1 in HCC tumors compared to non-tumor tissues [92]. Nevertheless, the Warburg effect of cancer cells often creates the false impression that cancer cells do not require mitochondrial metabolism [93]. It is increasingly becoming evident that cancer cells do possess active mitochondria and that oxidative metabolism has a role to play in tumor development. The protein levels of most TCA enzymes do not change in HCC when compared to neighboring liver tissue of the HBV-related cohort, although the majority are heavily lactylated [93]. In human and mouse HCC, expression of succinate dehydrogenase (SDH) is most commonly down-regulated and associated with a poor prognosis. SDH inhibition induces HCC formation through the accumulation of succinate, and via the Yes-associated protein (YAP)/transcriptional coactivator with a PDZ-binding domain (TAZ) pathway [93]. The inhibition of ATP synthase (complex V) by oligomycin in combination with the anti-glycolytic drug 2-deoxyglucose (an analog of glucose) promotes the death of breast cancer cells, which indicates that OXPHOS plays a key role in cancer progression [94]. Some HCC cell lines are also sensitive to OXPHOS inhibitors, based on their basal rates of mitochondrial respiration [95].

### 4.4 Changes in Fatty Acid Metabolism in TME

**Figure 3:**
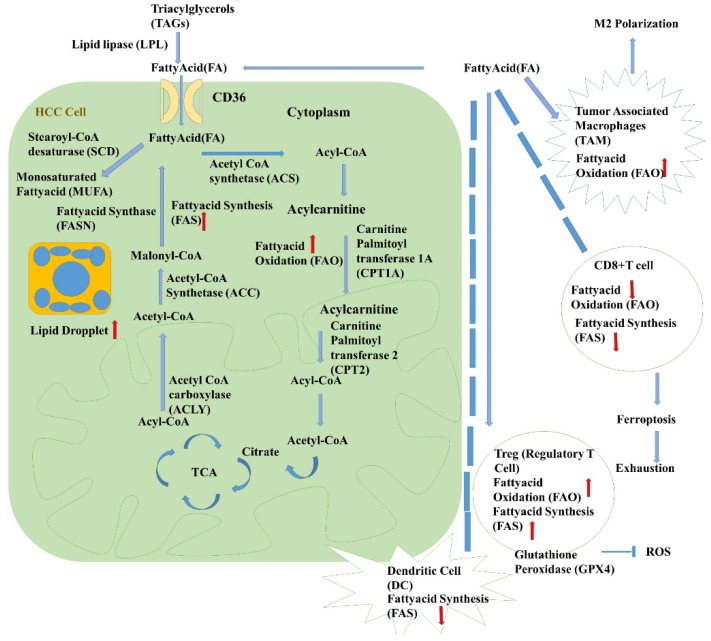
Competition of fatty acid uptake and reprogramming of lipid metabolism on the TME. M2 TAM polarization is facilitated by accumulated FAs. Decreased FA production worsens the work of DCs, interfering with the processing of antigens and the development of the immune response. High GPX4 levels in Tregs are characterized by an improved usage of nutrients and increased FAS and FAO rates, whereas the CD8^+^ T-cells die due to oxidative stress, resulting in T-cell exhaustion and ferroptosis. The dotted line shows lower uptake or a decreased effect of promotions. Where CD is cluster of differentiation 36. HCC is hepatocellular carcinoma, TME is tumor microenvironment, TAM is tumor-associated macrophages, FA is fatty acids, DCs are dendritic cells, GPX4 is glutathione peroxidase 4, FAS is fatty acid synthesis, FAO is fatty acid oxidation, and ROS is reactive oxygen species.

The gradual change of the liver tissue into HCC tissue with poor nutritional supply is accompanied by a shift in metabolism towards lipid dependence instead of glucose-dependent metabolism. Lipids may serve as secondary messengers to regulate signaling cascades such as Wnt and TGF-β, or carcinogenic transcription factors such as NF-κB, HIF-1α, AP-1, and STAT3 [96]. They are also capable of mediating post-translational changes. HCC TMEs are often characterized by disturbed lipogenesis and lipid accumulation, which have different influences on the immunological functions of cellular components. Lipid lipase (LPL) is upregulated in HCC and increases exogenous lipid transportation by converting triacylglycerols into fatty acids (FAs) [97] as depicted in Fig. 3. It also enhances the expression of the FA transporter CD36 [97]. Moreover, there is increased lipogenesis in HCC by enhanced levels of Acetyl-CoA carboxylase (ACC), fatty acid synthase (FASN), and ATP citrate lyase (ACLY) [98]. In CTNNB1-mutated HCC cells, oxidized FAs or elevated *de novo* FA synthesis are critical sources of energy to undergo metabolic reprogramming [99]. Liver inflammation is increased due to excess FAs by fatty acid synthesis (FAS). Accumulation of palmitic acid (PA) in Hep3B cells stimulates the release of TGF-β1 and CSF1, which supports immunosuppressive programming of cancer-associated fibroblasts and favors M2-type tumor-associated macrophage polarization [100]. Nevertheless, FAs have concentration-dependent effects on T-cells: high concentrations of FAs can cause malfunction and death by lipotoxicity, whereas low concentrations can modify proliferation [101]. ACC, SCD, and FASN are under the control of SREBP-1 and mTORC1, a mechanism that suppresses fatty acid generation in dendritic cells [102]. One of the most used blood biomarkers in the diagnosis of HCC is AFP (α-fetoprotein), which is produced by HCC cells and partially contributes to the downregulation of FA synthesis [103]. Overall, AFP-associated suppression of fatty acid production interferes with antigen processing and presentation cascades, thereby hindering immune response stimulation and the development of DCs [104]. Thus, the complex metabolic and immune regulatory network in HCC TMEs is evidenced by the targeted impact of dysregulated fatty acid metabolism on specific cellular elements. They also underline the importance of targeting with great accuracy in order to control immunological processes [104]. In addition, changes in the expression of carnitine palmitoyl-transferase (CPT), which transports Acyl-CoA to mitochondria, and dysregulated fatty acid oxidation (FAO) in TME cells are essential in metabolic remodeling [105]. FAO is a vital energy source of tumor cells with increased expression of CPT2 and peroxisome proliferator-activated receptors (PPARs) in β-catenin-activated HCC. However, IRE-1α (inositol-requiring protein) mediated lipid peroxidation exacerbates immunological dysfunction in DCs while increasing polarization of M2 macrophages [96]. Tregs, in turn, can increase FAS and FAO rates and are more efficient at utilizing nutrients because they express high levels of glutathione peroxidase 4 (GPX4) [96]. TILs (Tumor-infiltrating lymphocytes), however, cannot avoid oxidative stress, leading to ferroptosis and T-cell dysfunction. A fundamental element of inflammation and necrosis, RIPK3 (Receptor-interacting protein kinase 3), is suppressed in TAMs and enhances polarization of M2 [106]. The deficiency of RIPK3 decreases oxidative stress and caspase-1 activity, triggering PPAR activation and substantially increasing fatty acid metabolism [107]. Hence, the RIPK3-ROS-Caspase1-PPAR cascade facilitates fatty acid oxidation and M2 polarization of TAMs [108]. scRNA-seq data of human and mouse HCC identified a CD36^+^ cancer-associated fibroblast subset derived from hepatic stellate cells that promotes p38 signaling, recruits CD33^+^ myeloid-derived suppressor cells, and enhances lipid peroxidation [109].

### 4.5 Cholesterol Metabolism

*De novo* fatty acid synthesis, also known as *de novo* FAS, is one of the important ways that tumor cells acquire lipids. HCC cells have increased *de novo* FAS activity [96]. Two key enzymes of *de novo* FAS, ATP citrate lyase (ACLY) and fatty acid synthase (FASN), are overexpressed in HCC and have been associated with a poor prognosis [110]. Several transcription factors and signaling pathways regulate *de novo* FAS in HCC cells. Sorafenib leads to HCC cell death by disrupting monounsaturated fatty acid production mediated by stearoyl-CoA desaturase-1 (SCD1) through the ATP-AMPK-mTOR-SREBP1 cascade [111]. Impaired cholesterol production is also a feature of lipid metabolic remodeling in HCC [112]. In HCC cells, higher levels of mitochondrial cholesterol reduce the permeability of the mitochondrial membrane, obstructing the cytochrome c release and causing chemotherapeutic resistance [113]. SIRT4 is an ADP-ribosyl transferase, a Sirtuin family member that regulates the expression of both mitochondrial and FAO genes in muscle and liver cells [114]. In TAMs, reduced SIRT4 levels is link with an increase in transcription of fatty acid oxidation and mitochondrial genes such as CPT1, Cyt C, PDK4, and IDH3. Also, SIRT4 deficiency contributes to the development of HCC by enhancing polarization of M2 of TAMs via the PPARd-STAT3 signaling cascade [115].

### 4.6 Impact of Dysregulated Amino Acid Metabolism in the TME

**Figure 4:**
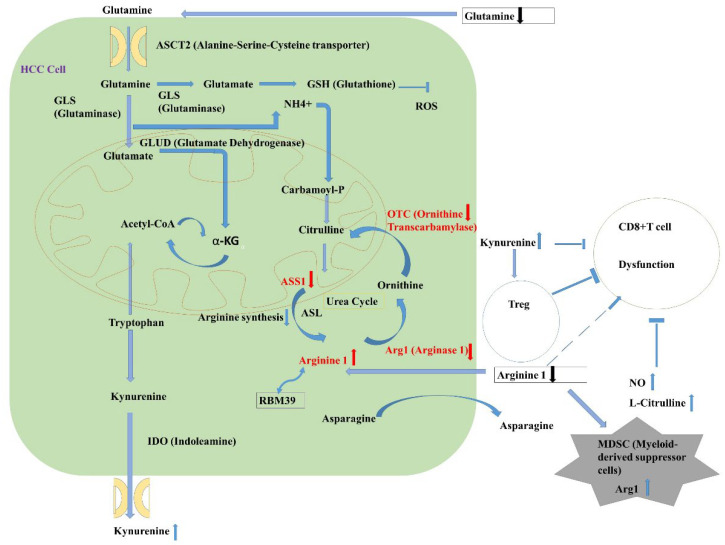
Effects of abnormal amino acid metabolism on the TME. HCC cells absorb glutamine in TME to sustain nucleotide and protein synthesis, and the generation of antioxidant GSH. The reaction of tryptophan to kynurenine mediated by IDO drains tryptophan and accumulates kynurenine, resulting in Treg production. Arginine synthesis is inhibited by a lack of ASS1 or OTC in HCC cell lines. Nevertheless, elevated levels of arginine in HCC cells caused by increased import of arginine and decreased expression of Arg1 lead to liver carcinogenesis. Increased arginine is bound to RBM39, which regulates metabolic gene expression and enhances the production of asparagine and the importation of arginine, forming a positive feedback mechanism in the development of cancer. Arginine is broken down into NO and L-citrulline by MDSCs, which exhausts nutrients of immune cells. Altogether, the immunosuppressive TME is induced by dysregulated amino acid metabolism. The dashed line indicates reduced uptake. Where TME is tumor microenvironment, HCC is hepatocellular carcinoma, α-KG is alpha-ketoglutarate, IDO is indoleamine 2,3-dioxygenase, ASS1 is argininosuccinate synthase 1, OTC is ornithine transcarbamylase, Arg1 is arginase 1, MDSCs are myeloid-derived suppressor cells, NO is nitric oxide, GSH is glutathione, ROS is reactive oxygen species.

Amino acids are another important source of metabolic substrates, supplying both carbon and nitrogen for protein and nucleotide biosynthesis and for maintaining redox balance. To facilitate the production of nucleotides, proteins, and the antioxidant GSH, the HCC cells take up the amino acids, glucose, or ions in the extracellular domain. This supplies energy to the TCA cycle in the case of hypoglycemia [116]. Fig. 4 demonstrates the impact of aberrant amino acid metabolism on the TME. HCC cells frequently exhibit glutamine addiction, driven in part by c-Myc-mediated upregulation of glutaminase (GLS) and increased glutamine uptake through transporters such as SLC1A5 (ASCT2) colonies. GLS1 generally promotes tumor growth and proliferation in HCC, whereas GLS2 has been reported to exert tumor-suppressive effects, partly through inhibition of PI3K/AKT signalling [117]. Moreover, the expression of GS and the synthesis of amino acids are further promoted by the positive feedback loop of Wnt, β-catenin, glutamine synthetase (GS), glutamine, and the mTOR/AKT axis [118]. This remodeling of the immune subpopulation is in line with metabolic reprogramming; the immunosuppressive type CD8-Tef-APOC2 has endogenous lipid and amino acid synthesis [119]. By contrast, the tumor-killing subpopulation CD8-Tef-GZMA requires exogenous lipids. Although glutamine metabolism is critical in tumor cells and CD8^+^ T-cells, glutamine disruption by HMGB1 inhibition enhances CTL penetration and improves immune response due to the high degree of metabolic flexibility of CTL [119]. Other amino acid metabolites, including arginine (Arg) and indoleamine 2,3-dioxygenase (IDO), produce immunosuppressive TMEs. IDO induces Treg production and promotes tryptophan deprivation and kynurenine accumulation by promoting tryptophan to kynurenine conversion [120]. Given the immunosuppressive effects of IDO-mediated tryptophan metabolism and arginine depletion in the TME, these pathways have also been investigated as promising therapeutic targets. Pharmacological inhibition of IDO or arginine depletion strategies aimed at modulating arginine metabolism have been investigated to restore effective T-cell activity and improve anti-tumor immune effects [121]. However, clinical translation remains under investigation; these strategies depict the therapeutic relevance of the amino acid metabolic pathway in HCC. Besides being a precursor of polyamines, nitric oxide, and creatine, arginine is a highly versatile type of amino acid that influences the metabolism of various cellular composites in HCC TMEs differently. Citrulline is first metabolized by ASS1 to form arginosuccinate, which is further metabolized to form Arg by arginosuccinate lyase (ASL) [118]. Arg1 subsequently breaks down Arg into ornithine, which OTC changes back to citrulline and is again converted back to Arg by ASS1/ASL [122]. An increase in the amount of Arg in HCC tumor cells, which propagates liver carcinogenesis, however, is induced by increasing the import of arginine and inhibiting the conversion of Arg to polyamine by suppressing the expression of Arg1 [123]. Elevated levels of Arg change metabolism by binding to RNA-binding motif protein 39 (RBM39), which transcriptionally regulates the expression of metabolic genes, such as the overexpression of the gene encoding asparagine synthetase (ASNS) [124]. Increased ASNS expression generates a positive feedback loop between high levels of Arg and active carcinogenic metabolism, and promotes the synthesis of asparagine and Arg importation [124]. MDSCs use elements to generate proteins by secreting Arg1 and nitric oxide synthase-2 (iNOS2), respectively, that further decompose L-arginine and L-citrulline into nitric oxide (NO) and L-citrulline [125]. It leads to insufficient provision of nutrients to the immune cells, impairment of transmission of JAK-STAT signals, inhibition of MHC class II expression, and inability to conduct immune surveillance [126]. Consequently, Arginine mediates metabolic remodelling and carcinogenesis and may function as a second messenger-like molecule [126]. Finally, the alteration of immunological functions and subpopulation remodeling is associated with dysregulated amino acid metabolism and expression of such metabolites as IDO and Arg, especially in c-myc or β-catenin-activated HCC [118].

### 4.7 Local and Systemic Metabolism

Anatomical location and overall metabolic condition of the patients significantly influence the metabolic landscape, besides cellular interaction in TME. Conversely, the latter is influenced by multiple systemic factors, including dietary factors and gut microbiota, whereas the former is determined by liver-specific metabolism and regional metabolic differences within hepatic tissue [127]. Oxygenated and nutrient-rich blood is delivered to the liver through the combined input of the hepatic artery and portal vein [2]. Post metabolic processing and synthesis of metabolites are delivered to the liver via the portal vein, exposing it to many GIT metabolites, and to the systemic circulation via the biliary system [128]. The immune condition of the TME depends on varying metabolic conditions that are supported by dissimilar perfusion and cellular structure [129]. Based on variations in metabolic functions, hepatocytes are broadly classified into periportal and perivenous subtypes. Enzymes in the processes of detoxification, glutamine production, and glycolysis are greatly enriched in perivenous hepatocytes. Moreover, the periportal area is characterized by a high concentration of the urea cycle and gluconeogenic enzymes [130]. This metabolic distribution reflects active zonal β-catenin and APC regulation in the periportal zone, contributing to enhanced glutamine production in nearby hepatocytes [131]. It is also accomplished by enhanced level β-catenin target genes such as Glt1 and Oat (ornithine aminotransferase) [132]. Urea synthesis after ammonia fixation is restricted to periportal hepatocytes, but this function is reduced in HCC and replaced by the production of glutamine, along with expanded GS expression in perivenous cells of liver tissues [133]. Nevertheless, additional studies are required to gain a deeper appreciation of the impact of the HCC metabolic region on the immune system and clinical outcome.

## 5 Biomarkers in Hepatocellular Carcinoma

### 5.1 Diagnostic Biomarkers

#### 5.1.1 Imaging and Tissue Biopsy

The AASLD guidelines endorse twice-yearly ultrasonographic monitoring for patients at high risk of HCC [134]. Ultrasonography is recommended for routine surveillance; however, it depicts modest sensitivity while retaining high diagnostic specificity [135]. However, the therapeutic window can be reduced because it recognizes early lesions and tumor nodules only to a limited extent [135]. Imaging (CT, MRI, and ultrasound) is often utilized to measure the number and size of hepatic tumor nodules [136]. CT and MRI are much more accurate in detecting HCC, with an accuracy of more than 90% in tumors larger than 2–3 cm in diameter. This transverse imaging is costly, even though it is highly sensitive and accurate, and can only be incorporated in the normal routine surveillance when clear indicators begin to appear [137]. Tissue biopsy is not common until the imaging findings confirm the diagnosis only partially [138]. As an example, a tissue biopsy may contribute to an accurate diagnosis when non-cirrhotic liver disease is suspected or when the lesion is small, and imaging fails to reveal it [139]. Further, a biopsy may provide information on genetic mutations directly in the tumor tissues and aid in the differentiation between the uncommon cancer types and HCC [140]. It is necessary for precision medicine. Nevertheless, tissue biopsy can be considered a surgical procedure that can cause liver damage, discomfort, and bleeding. Also, when tumor cells do not stay at the point of origin but move to other parts of the wound, there exists a low probability of inducing an intrahepatic metastasis. Tissue biopsy remains a vital means of diagnosing HCC after all, as it is capable of providing a definitive diagnosis using histological evidence. Pathologists are able to perform tumor staging more precisely, which benefits the patient. To monitor the progress of treatment and provide prognostic information, tissue biopsies can be conducted. Also, it helps build our knowledge about cancer biology, innovative biomarkers, and the creation of innovative treatments [141].

#### 5.1.2 Innovations in Liquid Biopsy Diagnostics

For prompt information and maximum feasibility in screening and response detection, liquid biopsy is now an important tool. Blood is the common fluid to be collected in a therapeutic setting. Nevertheless, other bodily fluids include bile, saliva, urine, and ascites fluids [142].

#### 5.1.3 Serological Diagnostic Biomarkers

##### AFP

The most prevalent serum protein marker is AFP, the expression of which increases during tumor progression. The level of AFP, a glycoprotein secreted in the fetus, the fetal analog of albumin, should decline after childbirth. This glycoprotein (MW 70 kDa) transports steroids, fatty acids, heavy metals, bilirubin, retinoids, flavonoids, phytoestrogens, dioxin, dyes, medications, and others [143]. It is produced by the liver of the fetus, the yolk sac, and the intestine in the course of development. Between week 12 and 16 of fetal growth, AFP in serum peaks (3 g/L) [144]. The levels then soon decrease to a point where traces can be found in serum only. Unusually high levels of AFP in serum are linked with malignant diseases, including HCC. Depending on the lectin binding pattern, AFP exists in three glycoforms: AFP-L1 (non-binding fraction), AFP-L2 (weak binding fraction), and AFP-L3 (the binding fraction) [145]. There is an enhanced level of AFP-L3 in HCC and AFP-L1 in chronic hepatitis and liver cirrhosis. The level of AFP-L1 is increased in liver cirrhosis and chronic hepatitis, whereas the level of AFP-L3 is significantly elevated in HCC. The AFP-L3 is regarded as a specific HCC biomarker since it is exclusively produced by cancer cells [146].

##### DCP

The inactive form of prothrombin is DCP (Des-gamma-carboxy prothrombin) or PIVKA-II protein (activated by antagonist II or in the absence of vitamin K) [147]. The loss of normal prothrombin activity by DCP helps to support malignant development in HCC. The level of DCP differs considerably between malignant and benign hepatic patients [148]. Compared with AFP, it could be a more sensitive diagnostic tool. This outcome still has to be validated. Moreover, DCP and AFP-L3 are not linked with high levels of total AFP, so they are the appropriate candidate markers to be utilized along with AFP to determine HCC [149]. Although AFP, AFP-L3, and DCP are widely used biomarkers in HCC, their clinical performance must be interpreted in conjunction with imaging modalities such as CT, MRI, and ultrasound. In most clinical settings, these serological markers give complementary rather than superior diagnostic value compared with imaging-based detection [150]. Moreover, their sensitivity for early-stage HCC remains limited, as a substantial proportion of early tumors may not produce a detectable elevation of these biomarkers. These limitations highlight the need for integrated diagnostic linking imaging techniques with serological and molecular biomarkers to improve early detection of HCC. In existing clinical practice, AFP, AFP-L3, and DCP are more attainable and better developed than liquid biopsy assays, making them a helpful addition for surveillance [151]. However, these markers do not give the tumor-specific genomic or vesicle-based information obtainable from ctDNA and EV analysis. Therefore, rather than replacing liquid biopsy platforms, serological biomarkers are more suitable when viewed as complementary tools that may support imaging-based surveillance and multifaceted diagnostic assessment [152].

##### GPC-3

A heparan sulfate proteoglycan is Glypican 3 (GPC-3), which is precisely linked with the growth of the tumor and performs a main role in proliferation and cell division. Recently, GPC-3 emerged as a specific target for HCC treatment and diagnosis [153]. Normal hepatocytes and diseased liver cells of cirrhosis and hepatitis rarely ever express GPC3. Conversely, GPC-3 is particularly heightened in the tissues of HCC. By increasing the autocrine/paracrine canonical Wnt signaling cascade, GPC-3 enhances the growth of HCC [153].

##### Golgi Protein-73 (GP73)

The Golgi complex contains the transmembrane glycoprotein GP73 (MW 73kDa). GP73 is found in biliary epithelial cells and not in normal hepatocytes, so it is highly expressed in liver disorders such as HCC [154].

#### 5.1.4 Circulating DNA-Based Metabolic Biomarkers

The term cell-free DNA (cfDNA) refers to the concentration of nucleic acids present in the blood that is usually shed off dead cells due to disease or cell turnover. cfDNA is present in normal persons, and its main source is blood cells [155]. All the cells of the peripheral blood are isolated in order to conduct the analysis of the cfDNA. Uncontrolled cell death in the tissues and organs of cancer patients discharges a significant amount of nucleic acids into the blood, which make up the ctDNA [156]. Analysis of ctDNA gives information about a diverse population of tumor cells that contains the epigenetic patterns and specific mutations, as ctDNA comes directly from cancer cells. Moreover, circulating tumor cells are phagocytosed by macrophages, which in turn release nucleic acids that take part in the pool of cell-free DNA [157]. Hence, ctDNA is termed the tumor-derived fraction of cfDNA, composed of fragmented DNA from tissue cancers. The proportion of ctDNA of total cfDNA may differ greatly, but may be below 1 percent to more than 90 percent.

In clinical practice, ctDNA is differentiated from background cell-free DNA through the identification of tumor-associated molecular changes. These changes may include somatic mutation, copy number variations, or mutated DNA methylation patterns, hallmarks of tumor cells [158]. Analytical thresholds are broadly established using variant allele frequency-based assay criteria or mutation-specific assays substantiated against matched tumor tissue samples. In next-generation sequencing technologies, ctDNA detection depends on identifying tumor-derived variants above the method-specific-sensitivity threshold, allowing reliable differentiation between tumor-derived ctDNA and non-tumor circulating DNA in patients with HCC [159].

Another parameter of tumor burden, ctDNA, is generally present in the blood in high concentrations in patients with HCC. Application of ctDNA in clinical practice has its challenges, like that of CTCs. The differentiation between ctDNA and a reservoir of cfDNA, which represents other cell types, is very difficult to perform [157,160]. Also, it remains unknown as to whether ctDNA is capable of accurately capturing the genetic composition of highly migratory and fast-growing tumor cells. This can be explained by the fact that ctDNA can disproportionately represent the DNA of the so-called attenuated tumor cells that are more likely to die and secrete their genetic elements into the bloodstream [161]. More research in this field is needed to fully exploit ctDNA as a monitoring tool. Relative to conventional serological biomarkers, ctDNA gives tumor-specific genetic and epigenetic information and may therefore provide augmented value for dynamic disease assessment and molecular stratification [162]. Nevertheless, its clinical use is currently impeded by low abundance in early-stage disease, operational intricacy, and the need for highly sensitive analytical frameworks. Hence, ctDNA is preferably considered as a complementary biomarker for molecular analysis and serial assessment rather than a direct substitution for established serum-based assessment tools [142].

#### 5.1.5 Circulating Vesicles Originating from Tumor Cells

EVs (extracellular vesicles) are small and membrane-encased vesicles that contain a wide range of cellular components. The three common classes of extracellular vesicles, exosomes, apoptotic bodies, and microvesicles differ in their size, molecular cargo, and mechanisms of release from cells [163]. EVs carry diverse biological molecules, including DNA, RNA, lipids, and proteins. EVs are involved in essential biological functions such as tissue remodelling and inflammation, and they are needed to support cell-to-cell communication. Some of the liver cells that are released into the blood include hepatocytes, hepatic stellate cells, and endothelial cells. Vesicles originating from the tumor cells are known as tumor-derived vesicles. Long non-coding RNAs (lncRNAs), which play important functions in the maintenance of genetic and cellular processes, have been identified within EVs and are linked with various diseases. Because the molecular composition of EVs can be changed by various cellular stress conditions, EVs have potential value as biomarkers for hepatocellular carcinoma (HCC) [164]. Relative to ctDNA, EVs may give a wide representation of tumor biology because they carry proteins, lipids, DNA, and multiple RNA species that represent active intercellular signaling within the TME. This attribute may offer benefits for identifying functional signaling variations [165]. Nevertheless, major challenges persist in EV isolation, purification, quantification, and platform harmonization, which at present limit their routine use as alternatives for either ctDNA-based assays or serological biomarkers in clinical settings.

Collectively, these biomarker classes are more supplementary than substitutable in the recent HCC setting. Serological markers such as AFP, AFP-L3, and DCP remain applicable for periodic assessment because of their availability and affordability [150], whereas ctDNA provides greater target specificity for tumor profiling and monitoring [166], and EV-based biomarkers may offer wider insight into biologically active signaling procedures [167]. Currently, nevertheless, no single liquid biopsy biomarker can fully replace the others across all clinical settings, and combined multimodal strategies will likely be needed for efficient detection, stratification, and therapeutic monitoring in HCC.

#### 5.1.6 miRNA and lncRNAs Related Biomarkers

Transcripts with no protein-coding regions are known as noncoding RNAs (ncRNAs). ncRNAs include long noncoding microRNAs (miRNAs) and RNAs (lncRNAs) [168]. In patients with HCV infection, non-invasive methods of early HCC diagnosis can include the identification of miR-221 and miR-101-1. Moreover, a set of these microRNAs was identified to yield high AUROC compared to AFP, with miR-519, miR-595, and miR-939 capable of distinguishing HCC and non-HCC in cirrhotic patients [169]. In a previous study, a miRNA panel (miR-122, miR-885-5p, miR-221, and miR-22) was reported to be capable of distinguishing between early HCC and liver cirrhosis, chronic hepatitis C, and normal individuals when the AFP was combined [170]. Consequently, it was proposed to be used clinically in the detection of HCC at its early stage, as the miR-16, AFP, AFP-L3, and DCP combination demonstrated a significant potential in HCC prognosis with sensitivity and specificity rates of 92.4 and 78.6, respectively. In the growth and development of HCC, lncRNAs have an important role. HCC-associated lncRNAs that are dysregulated in HCC tissues include HOTAIR, MVIH, HULC, PVT1, and MALAT1. As reports indicate, MALAT1 is highly sensitive in predicting human HCC [171]. In order to determine whether the above-mentioned lncRNAs can be effectively used in clinical practice, however, it is necessary to conduct more large-scale prospective studies.

#### 5.1.7 DNA Mutation and Methylation Related Biomarkers

Recent advances in the next generation have revealed detailed insights into the genetic landscape of HCC. An exome sequencing study of 243 hepatic cancer samples found mutations in the TERT (telomerase reverse-transcriptase) promoter as an early genomic event in HCC [172]. TERT promoter mutation could lead to the activation of telomerase or TERT expression, which might enhance the rapid growth and proliferation of tumors. The most frequent genetic alteration in HCC is TERT. However, enhancing GC levels in the TERT promoter zone inhibits the evolution of therapeutically useful tests to identify TERT promoter mutations in patient biopsies. Further studies on tumor DNA (cfDNA and ctDNA) and plasmatic cell-free DNA as a means of early detection of HCC by mutations in the TERT promoter are needed. HCC can be diagnosed using DNA methylation biomarkers, as epigenetic modifications, i.e., DNA hypomethylation and hypermethylation, are found as early stages in liver carcinogenesis [173]. A study involving patients with various cancer types found six HCC-specific hypermethylated CpG sites (cg11223367, cg05569109, cg11481534, cg23565942, cg03509671, and cg21908638 that depicted better diagnostic functions with specificities and sensitivities of 98% and 92%, respectively [174]. Altogether, for screening, diagnosis, and prognosing HCC, circulating tumor DNA methylation markers are regarded as trusted tools [148]. Despite the promising role of circulating tumor DNA mutation analysis and methylation-based biomarkers in hepatocellular carcinoma detection, several limitations currently restrict their routine clinical implementation. Analytical sensitivity remains a major challenge, particularly in early-stage disease, where tumor-derived DNA fragments are present at very low concentrations in circulation [175]. In addition, tumor heterogeneity may result in variable genetic and epigenetic alterations across different tumor regions and patients, potentially affecting the reliability of biomarker detection [176]. Furthermore, technical complexity, assay standardization, cost, and limited clinical validation continue to represent important barriers to the widespread integration of ctDNA-based and methylation-based diagnostics in routine clinical practice.

#### 5.1.8 Gut Microbiome Biomarkers

Gut microbial changes likely play a role via the microbiota-liver axis in liver disease and liver cancer, and the recognition of bacteria associated with HCC may be a valuable non-invasive predictor of liver cancer. In a prior comparison of the gut microbiomes of patients with HCC and NAFLD-related cirrhosis, the former had a decreased level of Bifidobacterium and an increased level of Bacteroides and Ruminococcaceae. Although Bifidobacterium deficiency can cause inflammation of the liver and intestine, and consequently impair the intestinal barrier [177]. Bacteroides and Ruminococcaceae have been associated with immune modification by inducing proinflammatory cytokines IL18 and IL13 and recruiting activated monocytes and mMDSCs. Oral microbiome can be used as a diagnostic biomarker of HCC along with gut microbiota. In the future, large-scale studies to determine the gut microbiome as a predictor of HCC risk are necessary [178].

### 5.2 Prognostic Biomarkers

Prognostic biomarkers provide information concerning disease progression, relapse risk, and overall survival in patients with HCC. Several tissue-based and circulating-based biomarkers have depicted prognostic significance in HCC. Increased serum AFP levels and constant AFP elevation after therapy have been associated with poorer survival outcomes and increased relapse risk [179]. Moreover, molecular changes detected in circulating tumor DNA, including TP53 and TERT promoter mutations, have been associated with aggressive tumor behaviour [180]. Epigenetic changes, such as aberrant DNA methylation patterns and gene expression signatures, have also been linked to disease progression [181]. These biomarkers may complement traditional staging systems, and facilities can improve risk stratification and long-term follow-up in patients with HCC.

### 5.3 Predictive Biomarkers

Predictive biomarkers are utilized to identify patients who are more likely to benefit from specific treatment strategies. In HCC, several molecular and immunological attributes have been investigated as potential predictors of treatment responses. For example, biomarkers linked to immune checkpoint signalling, including PD-L1 expression and the sequence of immune cell infiltration, have been investigated for their predictive value for response to immune checkpoint inhibitors [182]. Moreover, genomic changes found in circulating tumor DNA and metabolic pathway signatures may influence therapeutic sensitivity to treatment therapies and immunotherapy [183]. Although these predictive biomarkers are under investigation and require further validation in large clinical studies.

## 6 Treatment Interventions

**Figure 5:**
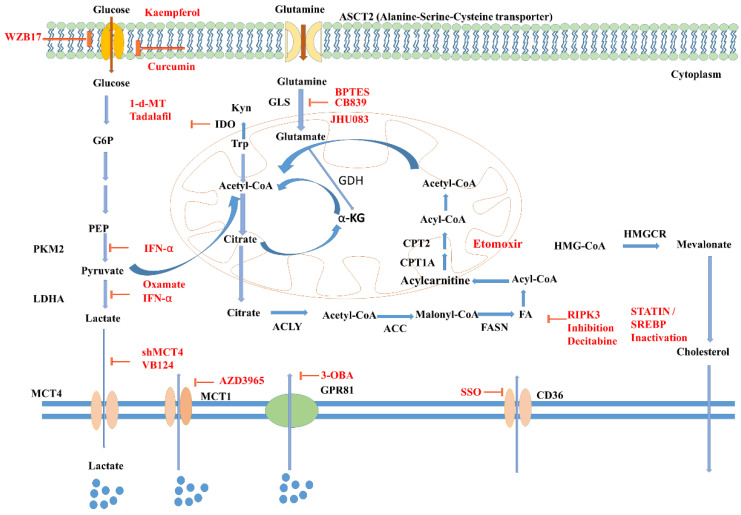
Stimulation of the immune system by attacking metabolism. The immune landscapes of HCC TMEs are remodeled by controlling the metabolic activities of dominant nutrients or enzymes. Where 1-d-MT is 1-methyl-d-tryptophan, ACLY is ATP citrate lyase, ASCT2 is alanine-serine-cysteine transporter 2, HMGCR is 3-Hydroxy-3-methylglutaryl-CoA reductase, IDO is indoleamine 2,3-Dioxygenase, Kyn is kynurenine, SREBP is sterol regulatory element-binding protein, PKM2 is pyruvate kinase isozyme type M2, CD36 is cluster of differentiation 36, CPT1A is carnitine palmitoyltransferase 1A, CPT2 is carnitine palmitoyltransferase 2, FA is fatty acid, FASN is fatty acid synthase, G6P is glucose-6-phosphate, GDH is glutamate dehydrogenase, GLS is glutaminase, HMG-CoA is 3-hydroxy-3-methylglutaryl-coenzyme A, HMGCR is 3-hydroxy-3-methylglutaryl-coenzyme A reductase, IDO is indolamine 2,3-dioxygenase, IFN-α is interferon-alpha, Kyn is kynurenine, LDHA is lactate dehydrogenase A, MCT1 is monocarboxylate transporter 1, MCT4 is monocarboxylate transporter 4, PEP is phophoenolpyruvate, PKM2 is pyruvate kinase M2, RIPK3 is receptor-interacting serine/threonine-protein kinase 3, SREBP is sterol regulatory element-binding protein, α-KG is alpha-ketoglutarate, SSO is sulfo-N-succinimidyl oleate, 3-OBA is 3-Oxobutyrate and Trp is Tryptophan.

Treatments targeting metabolic reprogramming in HCC are increasingly being explored to control tumor progression and immune evasion within TME. The approaches given below primarily focus on inhibiting key metabolic pathways that support tumor progression, modulating immune metabolic interaction within the TME, and enhancing the efficacy of immune checkpoint blockade through metabolic intervention. By targeting glucose, lipid, and amino acid metabolic pathways, several pharmacological agents and experimental techniques aim to disrupt tumor metabolic flexibility and restore antitumor immune response. The major metabolic pathways and therapeutic treatments involved in these techniques are illustrated in Fig. 5. Considering the variability of metabolic pathways involved in HCC progression, various metabolic inhibitors and modulatory techniques have been analyzed in recent years. In this review, exemplary metabolic inhibitors were selected based on their mechanistic significance to major metabolic pathways implicated in HCC, together with the availability of preclinical or clinical evidence reinforcing the therapeutic potential. Priorities were given to agents that have depicted efficacy in experimental HCC models, initiating early phase clinical testing, or revealed the ability to modulate tumor metabolism in combination with immunotherapeutic techniques. This approach enables the discussion to highlight inhibitors with the most pronounced translational significance instead of providing an exhaustive list of all documented metabolic compounds.

### 6.1 Direct Metabolic Inhibitors

Glucose transporter (GLUT) family proteins, particularly GLUT1, play a critical role in enhancing glucose uptake and glycolytic reprogramming in HCC. The uptake of glucose can be reduced by GLUT1 inhibitors, which have proven to block glycolysis, including kaempferol, WZB117, and curcumin [184]. In addition to glucose uptake, the export and reuse of glycolytic metabolites such as lactate are also necessary for sustaining tumor metabolic balance in HCC [185]. Moreover, monocarboxylate transporters (MCTs) are frequently overexpressed in cancer cells and are high-affinity transmembrane proteins [186]. They perform a main role in regulating intracellular pH and facilitate the transport of pyruvate, lactate, and ketone bodies. Among them, MCT4 mainly facilitates lactate export into TME, whereas MCT1 is mainly responsible for the uptake of lactate and pyruvate. On blocking MCT4 with VB124 or shMCT4, which is highly robust in inhibiting MCT4, tumor formation was evidently inhibited, and CTL infiltration and cytotoxicity, including glucose flow into the TCA cycle, were found to be enhanced [185]. Consistent with this logic, inhibition of lactate formation through targeting lactate dehydrogenase A (LDHA) has also been established as a therapeutic potential. In cell line-based studies, oxamic acid, which is an LDHA inhibitor, was shown to block the formation of lactate and act synergistically to improve the anti-cancer effects of other drugs, including sorafenib [187].

Beyond glucose metabolism, perturbed regulation of lipid metabolic pathways also mediates tumor progression and metabolic flexibility in HCC. Palmitoylation, regulated by palmitoyl-transferase such as ZDHHC17/24 mediates lipid signaling and retains oncogenic AKT activation in HCC cells. Restricting dietary palmitic acid intake or employing a FASN suppressor, orlistat, to inhibit the synthesis of palmitic acid after the TCA cycle, can significantly decrease the sustainment of palmitoyl modification and oncogenic AKT activity [188]. This could be because of compensatory upregulation of HMGCR expression (3-hydroxy-3-methylglutaryl-CoA reductase, a vital metabolic enzyme in cholesterol synthesis) and cholesterol biosynthesis, which can be countered by dual inhibition of FASN and HMGCR [189]. The proliferation of cells was inhibited by the use of siRNAs against FASN and HMGCR in a murine model of HCC, which is consistent with previous studies on the potential anti-tumor effect of statins [190].

Besides glucose and lipid metabolism, amino acid metabolic reprogramming, mainly glutamine metabolism, plays an important function in supporting tumor growth and metabolic plasticity in HCC. Inhibiting glutamine metabolism has been identified as an important therapeutic strategy. Small molecule inhibitors of glutaminase-1 (GLS1), including CB839 (BPTES analogs), ethyl sulfide (BPTES), and Bis-2-(5-phenylacet-amido-1,3,4-thiadiazol-2-yl), inhibit tumor growth by interfering with glutamine catabolism [191]. Nevertheless, most of these drugs have not been clinically tested due to poor solubility in water, low target specificity, and bioavailability. To overcome such concerns, prodrugs that selectively inhibit the metabolism of glutamine have been developed. A prodrug of this type is JHU083, a DON prodrug that is used to target glutamine in tumors with the use of nanocapsules [192]. The inhibition of glutamine metabolism with JHU083 in a subcutaneous tumor-forming mouse model increased the proliferation of CD8^+^ T-cells with reduced resistance to ICI [118]. These findings are derived from previously reported preclinical mouse model studies, in which experimental sample size and statistical analysis were defined within the original design. It was in part due to enriched exogenous lipid metabolism and lipolysis-generated ketone bodies that periodically replenished energy stores [118]. Glutamine addiction is a common property of tumors, especially when driven by mTOR stimulation and c-Myc mutations, which enhance the metabolic reliance of HCC cells on extracellular glutamine and glutamine catabolism [193]. Starving glutamine stimulates glutamate oxaloacetate transaminase 1 (GOT1) under the influence of C-myc and activates the Keap1-Nrf2 signaling pathway to increase GSH production to protect tumor cells against ferroptosis and ROS [194]. This suggests that combining GOT1 knockout with glutamine restriction can induce tumor cell death. The suppression of mTORC1 alone or together with Met in β-catenin-mutated HCC results in reduced tumor growth [195].

### 6.2 Immune Metabolic Modulator

Nanoparticles that are biocompatible in nature have been utilized as extracellular vesicles (EV) mimetics to increase metabolic communication within the tumor microenvironment. These EV-based techniques enhance the expression of PKLR in hepatocyte-derived EVs and to restore the level of fructose-1,6-bisphosphatase 1 (FBP1), a key gluconeogenic enzyme in recipient hepatocytes, in a diethylnitrosamine-induced mouse model of hepatocellular carcinoma (HCC) [196]. Restoration of FBP1 relieved gluconeogenic inhibition, reversed tumorigenic activity, and augmented NK cytotoxicity [185]. The immune-metabolite itaconate has also been widely examined for its role in modulating macrophage metabolism within the TME [197]. Low-dose ibuprofen (20 mg/kg) was shown in a mouse model of HCC to reduce the epigenetic interaction between macrophage metabolism and cytotoxic T lymphocyte exhaustion by inhibiting itaconate production and IRG1 expression, the enzyme responsible for itaconate synthesis [198].

Selective neutrophil ablation of IRG1 in Mrp8cre+IRG1f/fI mice sustained survival and delayed metastatic development. Moreover, deletion of IRG1 increased the therapeutic effects of ICB and restored T-cell anti-tumor activity. These observations suggest that immunometabolic targets such as itaconate and IRG1 may show promising modulators of antitumor immunity in HCC [199].

Beyond metabolite-based modulation, the metabolic profile of the TME itself can regulate immune responses through hypoxia-induced signaling pathways. The highly acidic and hypoxic environment activates the HIF-1α signaling, which reshapes immune modulation within the TME. TME. HIF-1α contributes to resistance to ICI by activating PI3K/AKT/mTOR signaling and promoting glycolysis [200]. However, IFN-α inhibits the transcription of HIF-1α and down-regulates glycolysis-associated gene expression through the activation of IRF1 and inhibition of downstream FosB transcription.

In addition to glucose mediated immunometabolic pathways, lipid metabolism also plays an important function in modulating immune cell polarization within the HCC microenvironment. Interleukin-4 (IL-4) induced polarization of macrophages toward the M2 phenotype can be reversed by suppressing fatty acid oxidation through CPT1A knockdown using shRNA or by treating with the clinically approved FAO inhibitor etomoxir [201]. Moreover, regulation of hypomethylation of RIPK3 by using decitabine or by targeting fatty acid oxidation in RIPK3 knockout mice to inhibit the metabolism of fatty acids increases the anticancer immune activity of TAMs and decreases polarization of M2. Despite the central role of fatty acids, other lipid classes like phospholipids also contribute to dysregulated lipid metabolism in HCC [202].

PLA2G7-high macrophages inhibit T-cell activation and have immunosuppressive effects within the TME. Pharmacological inhibition of PLA2G7 using darapladib induce NF-κB mediated M1 macrophage polarization and increases antitumor effects [203].

Amino acid metabolism represents another significant immunometabolic pathway that influences the activation of immune cells and therapeutic effects in HCC. Moreover, methionine supplementation enhanced the production of cytotoxic T lymphocytes producing TNF-α, IFN-γ, and IL-2, restoring immune functions in preclinical mouse models [204]. However, epigenetic RNA-RNA crosstalk between RICTOR and HMGB1 has been observed to modulate glutamine metabolism by stimulating GS (glutamine synthetase) and toll-like receptor 4 (TLR4) through the mTORC2–AKT–c-Myc signaling cascade [205]. RICTOR regulates glutamine metabolism and subsequently activates TLR4 and GS through the mTORC2-AKT-c-myc pathway [205].

Disruption of this signaling crosstalk inhibits mTORC1-dependent PD-L1 formation and decreases the production of PD-L1-containing exosomes, thereby inducing an immune response to ICIs in a murine HCC model [206].

Tadalafil (TA), an agent originally used to treat pulmonary hypertension and erectile dysfunction, has shown the ability to induce Arg1 deficiency in myeloid-derived suppressor cells (MDSCs) and repolarize macrophages toward an M1 phenotype, thereby suppressing resistance to ICI [207]. This resulted in the creation of a tumor-targeted nanocarrier that contained TA and anti-PD-1, and the subsequent administration of it in an orthotopic animal model of HCC. This nanocarrier reinstates CTL cytotoxicity by inhibiting the PD-1/PD-L1 axis [208].

### 6.3 Combination Approaches with Immune Checkpoint Inhibitors

While the previous section describes direct metabolic inhibitors and immune metabolic modulators individually, recent studies highlight that metabolic interventions can also be strategically combined with immune checkpoint blockade to control immune resistance and enhance therapeutic efficacy in HCC.

Immune checkpoint inhibitor resistance in HCC is widely recognized to be regulated by immunometabolic constraints within the TME. Rapidly proliferating cells compete with effector T cells for nutrients such as amino acids and glucose, hence limiting the nutrient resources needed for sustained T cell stimulation and cytotoxicity [209]. Simultaneously, tumor-derived lactate and TME acidification enhance immune suppression by attenuating T cell proliferation and cytokine expression while mediating checkpoint-linked tolerance [210]. Furthermore, exhausted T cells show mitochondrial impairment and redox imbalance, which in turn decrease their effector capacity and may compromise responsiveness to immune checkpoint blockade. These findings depict that metabolic attributes of the TME, including lactate build-up, nutrient competition, and T cell metabolic competence, may serve as predictive factors of treatment efficacy, although clinically established metabolic biomarkers continue to be constrained [211].

In this framework, combined treatment with interferon-α (IFN-α) and programmed death-ligand 1 (PD-L1) blockade reshapes the metabolism of glucose, with enhanced CD27 expression, increased cytotoxic activity, and a more effective anticancer immune response [200]. Similarly, combined blockade of PD-L1 and IFN-α results in substantial tumor regression, as demonstrated in mouse models and supported by clinical observations involving patients with HCC, receiving this combination therapy. In addition, lactate lowers intracellular cAMP and PKA through activation of the lactate receptor, GPR81, and HIF-1α signaling cascades, which subsequently leads to tumoral PD-L1 expression [212]. The antagonist of GPR81, 3-hydroxy-butyrate (3-OBA), combined with the PD-1/PD-L1 cascade, results in a significant increase in infiltration of cytotoxic T lymphocytes and secretion of IFN-γ in animal models, ultimately leading to tumor regression *in vivo* [213].

In addition to glucose metabolism, lipid metabolic pathways also control the responsiveness of tumors to immune checkpoint blockade. Coculture of CD36-positive cancer-associated fibroblasts (CAFs) with hepatocellular carcinoma (HCC) cells promotes tumor growth and metabolic adaptation [97]. Treatment with immune checkpoint inhibitors combined with sulfosuccinimidyl oleate (SSO), an irreversible inhibitor of CD36 translocase activity, effectively restores T-cell immunity [214]. This effect is partly attributable to the lower metabolic flexibility of tumor cells compared with metabolically adaptable T cells. Pharmacological inhibition of PLA2G7 has been shown to increase the susceptibility of HCC cells to immune checkpoint blockade, both *in vitro* and *in vivo* [203]. To promote uptake of tumor-associated antigens (TAAs) and overcome impaired immune effects resulting from poor antigenic internalization following combined ICB and thermal ablation, nanodrugs have been intratumorally administered in experimental studies [215]. The liberated tumor-associated antigens (TAAs) were engulfed by nanodrugs and eventually internalized by dendritic cells, combined with TAAs and an m6A demethylase inhibitor, also called a fat mass and obesity associated protein (FTO) inhibitor [216]. This nano-drug technique effectively increases maturation of DCs, enhances presentation of antigen, and strengthens anticancer immune response in a mouse model of HCC [216].

Amino acid metabolism represents another critical mechanism contributing to immune checkpoint resistance. In addition to glutamine metabolism, the kynurenine pathway also facilitates the immunosuppressive TMEs formation through upregulation of indoleamine 2,3-dioxygenase (IDO) mediated tryptophan catabolism, leading to kynurenine accumulation and the development of acquired immune resistance [205].

Targeting this pathway has therefore appeared as a putative therapeutic technique. Animal studies have demonstrated that 1-d-MT (1-methyl-d-tryptophan), an IDO suppressor, is capable of overcoming ICI resistance, which develops due to IFN-γ-induced upregulation of IDO in anti-CTLA-4-treated HCC cells [217].

As HCC cells are highly reliant on extracellular arginine and not on endogenous synthesis for survival, new therapeutic techniques have been developed to induce apoptosis or autophagy via arginine depletion. New studies have mainly focused on the utilization of arginine-degrading enzymes, including arginine deiminase and arginase1, to induce arginine depletion. Encouraging findings from early-phase clinical and preclinical studies using ADI-PEG 20 and recombinant human arginase 1 (rhArg1) have depicted that arginine depletion may help control resistance to ICI therapy [218].

Despite the promising preclinical and early clinical results discussed above, the effectiveness of combining metabolic strategies with immunotherapy may not be consistent across all HCC subtypes. Recent evidence suggests that tumors with immune-excluded phenotypes, especially those linked with the stimulation of the Wnt/β-catenin pathway, may show reduced responsiveness to immune checkpoint blockade despite therapeutic targeting of the metabolic pathway [219]. Moreover, several findings have indicated that the benefit of immunotherapy may differ according to the etiology of disease, with on-viral or NAFLD/NASH-related HCC depicting few consistent effects in some analyses, although these findings remain under investigation [220]. These contradictory findings show that the success of metabolism-based combination treatments is expected to rely on tumor-intrinsic molecular attributes, immune settings, and underlying hepatic disease, highlighting the need for subtype-specific therapeutic stratification in HCC.

### 6.4 Targeting Systemic Metabolism

The interactions among food, gut microbiota, and systemic metabolism exert significant effects on HCC development due to the close functional and anatomical relationships between the gut and the liver. Metabolic constituents derived from dietary intake enter the systemic circulation and interact with metabolic and immune receptors, hence shaping the TME [221]. In epidemiological studies, it was observed that obesity and excessive calorie intake are associated with an increased risk of tumor growth, whereas a low-fat and low-carb diet is linked with a reduced occurrence of tumors. Although dietary intervention could affect survival and prognosis, nutritional restrictions alone are unlikely to induce tumor regression, possibly because these interventions primarily effect increase cellular growth rather than tumor survival pathways [222]. Dietary modifications are believed to act by altering the functional states of cellular components and treatment outcomes, altering the availability of nutrients, and regulating metabolic states in systemic circulation and the TME. The following are some typical types of dietary intervention. Ketogenic diets (KDs) are characterized by high-fat and low-carb intake, which alter the availability of nutrients across systemic compartments and promote the utilization of ketone bodies as alternative energy sources. By reducing blood glucose levels and modulating insulin signaling, KDs have been found to be potential metabolic therapeutic techniques in cancer [223]. Moreover, genetic context may affect the response to ketogenic interventions. For example, KD-induced ketogenesis causes the development of BRAF V600E melanoma, enhanced BRAF signaling, and the accumulation of ketone bodies. A systematic review indicated that 10 of 24 clinical studies (42%) demonstrated supporting evidence of the beneficial effects of KDs in cancer prevention, whereas one study reported adverse effects, and seven studies observed no significant changes. Beyond macronutrient composition, dietary lipid profiles and metabolism of cholesterol also promote systemic alterations that influence HCC development. Moreover, mice fed a high-fat, low-cholesterol diet did not develop NAFLD-associated HCC, and instead gradually recovered from hepatic steatosis, whereas mice receiving a high-fat, high-cholesterol (HFHC) diet developed NAFLD-associated HCC and insulin resistance [224]. Cholesterol-driven HCC progression is associated with the intestinal microbiota dysbiosis, which enhances hepatic ROS accumulation, triggers intrahepatic natural killer T (NKT) cell infiltration, and increases the formation of pro-inflammatory cytokines, collectively resulting in tumor initiation and progression [225].

These findings highlight the critical function of the gut-liver axis in systemic metabolic regulation during HCC progression. Studies utilizing germ-free mouse models have revealed increased hepatic inflammation, cell proliferation, and lipid buildup following HFHC dietary exposure. Nevertheless, anti-cholesterol drugs such as atorvastatin have been shown to mitigate microbial dysbiosis and reduce NAFLD-associated HCC progression, indicating that cholesterol metabolism contributes to liver tumorigenesis through modulation of the gut microbial ecosystem [226]. The gut microbiota produces diverse metabolites that shape the immunological response and metabolism of cells, and these effects can be controlled with dietary intake [227]. The microbial taxa mentioned in this section were emphasized based on their reproducible association with HCC progression, immune regulation, or immunotherapy effects across several experimental and clinical findings. Special attention was given to microbial groups that have depicted consistent enrichment or depletion in HCC patients compared with healthy controls, in addition to taxa that have functionally associated with immune modulation, inflammation, or metabolic signalling pathways pertinent to tumor growth.

Moreover, factors observed to influence the effectiveness of immune checkpoint treatment in cancer cohorts were emphasized because of their putative translational values as predictive microbial biomarkers. When fecal samples from patients on ICIs were analyzed, microbial richness and gene counts were higher among responders. In patients with advanced HCC, differences in the microbial composition were also observed between responders and non-responders. Studies have revealed that melanoma patients who responded to ICIs showed higher Ruminococcaceae and greater fecal microbiota variability. Consequently, fecal microbiota transplantation from responder into mice enhanced T-cell responses, better efficacy of immunotherapy, and promoted better tumor control [228]. Hence, therapeutic modulation of the gut microbiota has been found to be a potential technique to improve immunotherapy effects. Targeting the gut microbiota via approaches such as FMT (fecal microbiota transplantation), antibiotics, or probiotics is under investigation, as the interaction between the microbiota and immune checkpoint signaling may contribute to immune evasion. The combination of FMT with PD-1 inhibitors has been observed in several clinical studies to assess its safety, feasibility, and therapeutic effects [229]. Following fecal microbiota transplantation from responders and the re-initiation of anti-PD-1 therapy, a full response and two partial responses were observed in the clinical study [230]. Probiotics may also maintain the intestinal barrier integrity, maintain the homeostasis of gut microbes, and prevent intestinal microbial metabolites from migrating and leaking into the bloodstream [231].

Despite these findings, the mechanism through which dietary modulations and microbiota-associated systemic metabolism influence immunological effects remains incompletely understood. Further investigations are needed to evaluate how systemic metabolic intervention can be effectively integrated with immunotherapeutic strategies in HCC.

Although these approaches show the therapeutic potential of systemic metabolic intervention in HCC, their implementation into routine clinical practice remains limited by biological heterogeneity and patient-dependent variability.

### 6.5 Translational Barriers and Clinical Considerations

Despite the promising preclinical findings of metabolic intervention in HCC, there are a few translational barriers that limit their routine clinical application. One major challenge is the metabolic plasticity of tumor cells, which enables cancer cells to adapt to metabolic burden by switching between alternative nutrient sources and metabolic pathways [232]. Hence, inhibition of a single metabolic pathway, such as glucose, lipid, or amino acid metabolism, may lead to adaptive metabolic reprogramming that constrains therapeutic effects [233].

Another important target is systemic toxicity. Many metabolic pathways targeted in cancer therapy are also necessary for normal tissue stability and immune cell function. Hence, therapeutic strategies that alter tumor metabolism may also impair the metabolic fitness of immune cells, likely reducing antitumor immunity [234]. Moreover, the heterogeneous metabolic landscape of HCC further impairs clinical translation [235]. Tumor metabolism exhibits significant variation depending on genetic changes, disease etiology, hepatic function, and the composition of the TME [236]. As discussed in Section 6.1, Section 6.2, Section 6.3 and Section 6.4, strategies targeting direct metabolic inhibitors, immune metabolic modulators, combination therapies with immune checkpoint inhibitors, and systemic metabolism modulation may provide variable therapeutic effects among patients.

Interventions targeting systemic metabolism, including cholesterol and dietary regulation, and modulation of the gut microbiota, further show the importance of host-related metabolic heterogeneity and influence immune responses and treatment effects [237,238]. Though these techniques may reshape immune effects and modulate treatment sensitivity, their clinical responses are difficult to regulate owing to the inter-individual variations in nutritional status, microbiota composition, metabolic background, and hepatic disease etiology [178].

Therefore, effective clinical implementation of metabolic techniques in HCC will need reliable biomarker-based patient stratification, improved interpretation of tumor and host metabolic heterogeneity, meticulously designed clinical trials that incorporate metabolic endpoints with immunological and therapeutic effects. A more well-defined framework linking metabolic susceptibilities with immune milieu and treatment selection will be essential for developing effective, safe, and personalized therapeutic techniques in HCC.

## 7 Conclusion

One of the key mechanisms of hepatocellular carcinoma evolution, immune evasion, and resistance to drugs is metabolic reprogramming. Altered glucose, lipid, and amino acid metabolism reshapes the tumor microenvironment and offers a source of clinically relevant biomarkers that may support metabolic stratification of HCC. By combining evidence on metabolic reprogramming, biomarker validation, and emerging therapeutic techniques, this review provides a comprehensive model for understanding how immunometabolic variations shape HCC progression and therapeutic effects. Increasing evidence demonstrates that metabolic pathways show important therapeutic susceptibilities in HCC, especially when metabolic modulations are rationally linked with immunotherapy to enhance antitumor immunity. However, the significant metabolic plasticity of HCC cells enables tumors to switch between nutrient sources and metabolic pathways, suggesting that targeting a single metabolic pathway may not always elicit durable therapeutic effects. Despite these advances, several fundamental questions remain unresolved, especially pertaining to how metabolic heterogeneity among HCC subtypes impacts therapeutic effect and how consistent metabolic biomarkers can guide patient stratification. Future research should clarify how metabolic variability across HCC subtypes modulates therapeutic effects and should identify reliable biomarkers capable of guiding metabolic stratification and patient selection. From a translational perspective, comprehensive metabolic profiling with genomic, immunological, and clinical data will be necessary for improving precision medicine approaches. Moreover, carefully designed clinical trials are needed to determine how metabolic interventions can be effectively combined with immunotherapy and other systemic treatments. Addressing these priorities will be critical for translating current knowledge of metabolic reprogramming into effective and personalized treatments for HCC.

## Data Availability

Not applicable.
